# Optimization of green biosynthesized visible light active CuO/ZnO nano-photocatalysts for the degradation of organic methylene blue dye

**DOI:** 10.1016/j.heliyon.2020.e04896

**Published:** 2020-09-23

**Authors:** Amr Fouda, Salem S. Salem, Ahmed R. Wassel, Mohammed F. Hamza, Th.I. Shaheen

**Affiliations:** a Botany and Microbiology Department, Faculty of Science, AL-Azhar University, Nasr City, Cairo 11884, Egypt; b Electron Microscope and Thin Films Department, Physics Research Division, National Research Centre, Dokki, Giza, 12622, Egypt; c Guangxi Key Laboratory of Processing for Non-ferrous Metals and Featured Materials, School of Resources, Environment and Materials, Guangxi University, Nanning 530004, China; d Nuclear Materials Authority, POB 530, El-Maadi, Cairo, Egypt; e National Research Centre (Scopus affiliation ID 60014618), Textile Research Division, (former El-Tahrir str.), Dokki, P.O. 1C2622, Giza, Egypt

**Keywords:** Materials science, Materials chemistry, Nanotechnology, Biosynthesis, Nanocomposites, *Penicillium corylophilum*, CuO, ZnO, Photocatalyst

## Abstract

Herein, CuO/ZnO nanocomposites at different ratios were successfully synthesized through a green biosynthesis approach. This was performed by harnessing the fungal-secreted enzymes and proteins during the sol-gel process for nanocomposites seed growth. All fabricated nanoparticles/nanocomposites were characterized using Fourier Transform Infra-Red (FT-IR) Spectroscopy, X-Ray Diffraction (XRD), Transmission Electron Microscopy (TEM), Scanning Electron Microscopy (SEM-EDX) and X-ray Photoelectron Spectroscopy **(**XPS) analyses. The photocatalytic degradation efficacy of the synthesized nanocomposites was evaluated using a cationic methylene blue (MB) dye as a model of reaction. Results obtained from the FT-IR and EDX analyses revealed that CuO-NPs, ZnO-NPs, CuO/ZnO_50/50_, CuO/ZnO_80/20,_ and CuO/ZnO_20/80_ were successfully prepared by harnessing the biomass filtrate of *Penicillium corylophilum* As-1. Furthermore, XRD and TEM revealed the variation in the particle size of the nanocomposites (10–55 nm) with the ratio of the nanoparticles. Notably, the size of the nanocomposites was proportionally increased with an increasing ratio of ZnO-NPs. XPS analysis affirmed the presence of both Cu and Zn in the nanocomposites with varying binding energies compared with individual nanoparticles. Furthermore, a high photo-degradation efficacy was achieved by increasing the ratio of ZnO-NPs in the nanocomposite formulation, and 97% of organic MB dye was removed after 85 min of irradiation using the CuO/ZnO_20/80_ nanocomposite.

## 1 Introduction

The startling technological development has increased the large-scale industrial production of much of the daily-used products with a broad range of applications [1, 2]. Among these products, dyes are crucial in the textile and fashion industries. However, with intensive applications, dyes transform into dangerous pollutants when they are improperly handled and disposed of causing serious environmental and public health hazards [3]. The common problems faced by textile industries are related to the residual dyes in the dyeing bath. Such industries implement innovative, rapid, and sustainable treatment methods to enhance the color removal of wastewater [4, 5]. Furthermore, legislation and laws related to the limits of discharge and reduction in the removal of colors are increasingly stringent [6]. Therefore, it is necessary to apply efficient treatment methods such as physical separation (membrane filtration), and chemical treatments, to reduce dye contaminants, organic, and inorganic pollutants. However, the operation cost and maintenance costs of these methods are considered as the basic and determining factors for large-scale applications [7]. Thus, inexpensive, efficient, eco-friendly and cost-effective alternative technologies are desired to minimize the problems of treatment liquid dye wastes [4]. Recently, photocatalytic degradation has proved to be a promising technique for the removal of organic dye pollutants by optical catalyst owing to its environmental friendliness and the absence of secondary pollutants [8, 9].

In recent years, nanomaterials in their different forms, shapes, and sizes have been discovered to be efficient in the removal of dye contaminants through photocatalytic activities [10, 11]. This is attributed to their unique physicochemical properties such as their structures, high mechanical strength, high width-to-height ratio, high thermal and electrical conductivities, slight advantage metal/semi-metallic weight and behavior, and high surface area [12, 13, 14, 15, 16]. Various types of nanomaterials, such as copper, zinc, and titanium, are employed in various treatments of dyes, including precipitation, decolorization, adsorption, and photo-degradation, as well as in the treatment of textile waste dyes [17, 18, 19].

Metal oxide nanocomposites, in particular, have been widely utilized in environmental research due to their huge applications, including catalysis and sensing applications [20, 21]. Among these nanocomposites, copper/zinc oxides (CuO/ZnO) have gained more interest in these applications owing to their optical, electrical, and magnetic properties, besides their eco-friendliness and excellent tunable catalysis characterization [22, 23, 24].

The main textual features obtained when two metal oxides semiconductors are coupled include high thermal stability and high surface area, which accelerate their reaction by enabling more active sites on their surfaces [25]. Also, this coupling induces mass and electron transfer without photo-corrosion of the nanocomposites and improves their efficacy [26]. Therefore, CuO/ZnO nanocomposites have a broad scope of bio-applications due to their physical, chemical, and low toxicity properties [8, 27].

CuO/ZnO of different sizes and shapes have drawn much interest compared to individual nanoparticles, especially CuO-NPs, which possess lower photocatalytic activities than ZnO-NPs [28]. The photocatalytic performance of CuO/ZnO nanocomposites is highly dependent on their surface area, size, and morphology, which can be varied by the method of preparation [8]. Various methodologies have been developed using chemical, physical, and biological approaches to produce ZnO-NPs, CuO-NPs and/or their coupling nanocomposites of different morphologies and shapes [4, 29, 30, 31]. Nanoscale sizes and shapes are the main factors that influence the performance of CuO/ZnO nanocomposites during the photolysis processes [32]. Among various methods of preparation, sol-gel is commonly used for the precipitation of CuO-NPs and ZnO-NPs, as well as their nanocomposites [33].

Compared with chemical and physical methods of synthesis, the green biosynthesis of NPs *via* biological processes is a greatly important and promising technique owing to its multiple advantages, including the use of natural products, safety, inexpensiveness, and environmental friendliness [12, 34, 35, 36, 37]. However, only a few studies have been reported the use of fungal metabolites in the synthesis of ZnO/CuO nanocomposites.

In this study, we focused on the biosynthesis of CuO/ZnO nanocomposites by harnessing the cell filtrate of *Penicillium corylophilum*. In addition, we developed various combinations with different ratios (ZnO/CuO_50/50_, ZnO/CuO_20/80,_ and ZnO/CuO_80/20_) to optimize the effect of the metal oxide ratios on the photocatalytic degradation performance of the composites. The fabricated nanostructures were characterized by XRD, FT-IR, TEM, SEM-EDX, and XPS. Finally, the biosynthesized nanostructures were employed in the photocatalytic degradation of methylene blue (MB) dye.

## 2 Experiment

### 2.1 Materials and methods

Copper acetate monohydrate [Cu(CH_3_COO)_2_.H_2_O] and zinc acetate dihydrate [Zn (CH_3_COO)_2_.2H_2_O] were purchased from Sigma Aldrich Company. They were used as the precursor for the biosynthesis of CuO-NPs, ZnO-NPs, and their composites. MB (C_16_H_18_ClN_3_S), which was used as a model for photo-degradation efficiency, was also purchased from Sigma Aldrich Company.

### 2.2 Fungal strain used for NPs synthesis

*Penicillium corylophilum* As-1 was isolated from a soil sample and cultured in a potato dextrose agar medium. The fungal strain was identified based on morphological and cultural examinations [[38], [39]], as well as molecular identification according to sequence analysis of internal transcribed spacer (ITS), as previously described by Salem et al [40], and its sequence was deposited in a gene bank under the accession number MN749557.

### 2.3 Nanoparticles synthesis

• **Preparation of biomass filtrate**; A disk (1.0 mm thick) of the cultured *Penicillium corylophilum* As-1 was inoculated in 100 mL of potato dextrose broth (PDB) media for 5 days at 30 ± 2 °C and 150 *rpm.* After the incubation period, the solution was filtered to harvest the fungal biomass, during which it was washed thrice with sterilized distilled water to remove any adhered impurities. About 10 g of the collected fungal biomass was mixed with 100 mL distilled water while stirred at 150 *rpm*, after which the mixture was incubated at 30 ± 2 °C for 24h [41]. Thereafter, the mixture was centrifuged at 5000 *rpm* for 10 min to separate the supernatant (biomass filtrate), which was later used for the green synthesis of CuO-NPs, ZnO-NPs, and CuO/ZnO nanocomposites as follow:
• **Biosynthesis of CuO-NPs**; One gram of Cu(CH_3_COO)_2_.H_2_O was dissolved in 1 mL distilled water, which was added drop-wisely to 99 mL of the biomass filtrate to get a final concentration of 5 mM. The pH of the solution was adjusted to 10 using a NaOH solution, which was added drop-wisely while the solution was being stirred [31]. The produced greenish precipitate was collected by centrifugation, washed twice with distilled water, and then oven-dried at 80 °C for 48 h.
• **Biosynthesis of ZnO-NPs**; 1.095 g of Zn (CH_3_COO)_2_. 2H_2_O was dissolved in 1 mL distilled water and then, mixed with 99 mL biomass filtrate to obtain a final concentration of 5 mM. The pH of the mixture was also adjusted to 10 using the NaOH solution, which was added drop-wisely while stirred. The white precipitate was collected *via* centrifugation and washed twice with distilled water after which it was oven-dried at 80 °C for 48h [42].
• **Preparations of nanocomposites**; To prepare the CuO/ZnO_50/50_, CuO/ZnO_80/20_ and CuO/ZnO_20/80_, a mixture of 0.5 g Cu(CH_3_COO)_2_.H_2_O + 0.55 g Zn (CH_3_COO)_2_. 2H_2_O; 0.8 g Cu(CH_3_COO)_2_.H_2_O + 0.219 g Zn(CH_3_COO)_2_. 2H_2_O and 0.2 g Cu(CH_3_COO)_2_.H_2_O + 0.876 g Zn (CH_3_COO)_2_. 2H_2_O were dissolved, respectively, in 20 mL distilled water. The respective mixtures were added drop-wise into 80 mL of the biomass filtrate, resulting in a final concentration of 5 mM. The pH of each composite solution was adjusted to 10 using the NaOH solution, which was added drop-wise while being stirred [43]. The formed precipitates were collected and washed twice with distilled water, followed by oven-drying at 80 °C for 48 h.

### 2.4 Characterizations

• X-ray Diffraction (XRD) Patterns.

The crystallinity of the biosynthesized CuO-NPs, ZnO-NPs, and their composites were studied by X-ray diffraction (XRD, X'Pert Pro Philips) using CuKα radiation, λ = 1.540 A^0^ (Eindhoven, Netherlands). The 2*θ* angle was from 0^o^ to 90^o^. The voltage and current were adjusted to 40 kV and 30 mA, respectively. The average particle size of each of the synthesized NPs was calculated using the following the Debye–Scherrer equation [44], shown below:

(1) *D* = *Kλ / βCosθ*, →

where D is the mean particle size and K is the Scherrer's constant (equal to 0.9). λ, β, and θ are the X-ray wavelength, Full-Width Half Maximum, and the Bragg's angle, respectively.

• Fourier Transform Infra-Red (FT-IR) Spectroscopy

The functional groups in the biosynthesized nanoparticles and nanocomposites were investigated *via* FT-IR analysis. The groups in the biomass filtrate responsible for the reduction, stabilizing, and capping of the nanoparticles were also investigated. The FT-IR analysis was performed using the Agilent system Cary 630 FT-IR model over a range of 4000–400 cm^−1^.

• Transmission Electron Microscopy (TEM)

The microstructural characteristics of the biosynthesized NPs, including size and shapes, were investigated *via* TEM analysis (JEM-1230, JEOL, Japan) at an operating voltage of 200 kV. A drop of the NPs colloidal solution was put on the carbon-coated copper grid. The excess of the colloidal solution was removed using a blotting paper. The loaded grid was dried at room temperature and placed directly in the grid box [45].

• Scanning Electron Microscopy (SEM-EDX)

The surface morphology and elemental structures of the biosynthesized NPs were analyzed *via* SEM (type: JEOL, JSM-6360LA, Japan). The SEM instrument was equipped with an energy dispersive spectroscope (EDX) to detect the surface shape and elemental compositions of different the NPs [46].

• X-ray Photoelectron Spectroscopy (XPS) Analysis

ESCALAB 250XI^+^ (Thermo Fischer Scientific, Inc., Waltham, MA, USA) equipped with a monochromatic X-ray Al Kα radiation (1486.6 eV) was used for the XPS analysis. For the analysis, the samples were prepared under the pressure of 10^−8^ mbar and the energy was calibrated with an Ag 3d_*5/2*_ signal (ΔBE: 0.45 eV) and C 1s signal (ΔBE: 0.82 eV). The size of the spot was 500 μm and the full and narrow-spectrum pass energies were 50 and 20 eV, respectively [47].

### 2.5 Photo-degradation efficiency

A solution of the photocatalyst was illuminated by the Heber Visible Photo reactor (Annular Type) equipped with 500 W tungsten halogen lamps. The light source was carefully localized on the solution during treatment to prevent light dispersion. Also, the light intensity was verified regularly by a lux meter to obtain the best light scattering in the photocatalyst process. An aliquot of the prepared samples was withdrawn at a predetermined irradiation time interval and analyzed at a λ_max_ = 664 nm for the cationic MB dye.

The photocatalytic reduction of the MB dye was investigated using 40 mg of the synthesized preliminary photocatalyst, which was mixed with 10 mL of the basic MB dye solution (10 mg. L^−1^) and subjected to irradiation under the effect of visible light at room temperature. Thereafter, 3.0 mL of H_2_O_2_ (0.08 M) was added and removed after intervals of irradiation time to extend the degree of decolorization. All the catalyst were carefully removed by external magnetic separation after centrifugation. The mixture was then stirred in the dark state in the batches for at least 130 min to ensure adsorption saturation before exposure to the light source. After saturation, about 3.0 mL of the suspension was collected and separated, and the absorbance of the supernatant was studied using a spectrophotometer. The diffused reflection for the synthesized nanocomposites as a function of wavelength for the ingot powder was recorded at an ambient atmosphere of 300 K by an optical spectrophotometer (Jasco model V-570) in the spectral wavelength ranged of 400–2000 nm. In the batch degradation experiment, the extent of dye removal in terms of percentage degradation was calculated as follow:

(2) *Degradation rate* % = *C*_0_–*C*_*t*_/*C*_0_×100,

where C_0_ is the initial dye concentration (mg. L^−1^) and C_t_ the dye concentration (mg. L^−1^) after time t (min).

### 2.6 Statistical analysis

All results presented in this study are the mean of three independent replicates. The data were subjected to analysis of variance (ANOVA) using the SPSS v17 statistical package. The mean difference between the treatments was analyzed by the Tukey HSD test at a significant level of *P* ≤ 0.05.

## 3 Results and discussions

### 3.1 Biosynthesis of CuO-NPs, ZnO-NPs, and their nanocomposites

**Figure 1:**
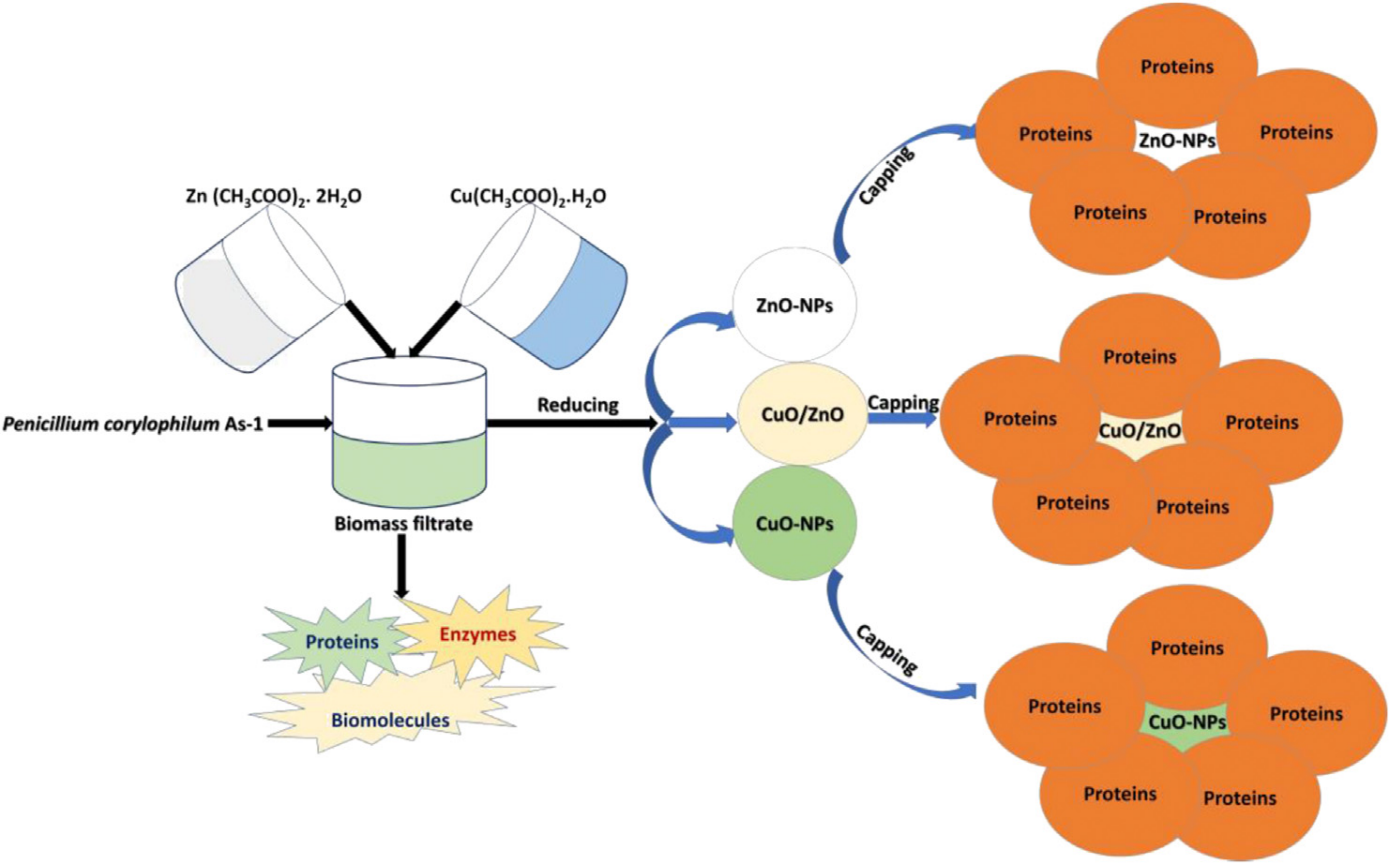
Flowchart showing biosynthesis of ZnO-NPs, CuO-NPs and nanocomposites using biomass filtrate of *P. corylophilum* As-1.

The fabrication of the NPs was dependent on the reduction and capping agents, which are the proteins and enzymes present in the biomass filtrate of the *P. corylophilum* As-1 (Figure 1). The capping agent played a critical role in preventing the formation of hydrated ZnO and CuO from uncontrollable agglomeration during the precipitation (Eq. 3).

(3) $$Zn^{+2}\text{/}Cu^{+2}\frac{proteins\;in\;biomass\;filtrate}{\Delta NaOH}Zn\left(\text{II}\right)O+Cu\left(\text{II}\right)O$$

Zn and Cu have the same valance in the used salts as well as in the obtained ZnO and CuO nanoparticles, hence, both ZnO and CuO nanoparticles are reduced to their nano-size using a capping agent. In this study, the purified structures of ZnO-NPs and CuO-NPs were synthesized *ex-situ*. Also, in the nanocomposites, all the nanoparticle were grown at the same time and shared the formed clusters with each other to form the nanocomposites. This interaction could be attributed to an intra-oxygen-metal-oxygen bond replacement such as –O-Zn-O-Cu-O-.

### 3.2 Characterizations of CuO-NPs, ZnO-NPs and their nanocomposites

• XRD patterns

**Figure 2:**
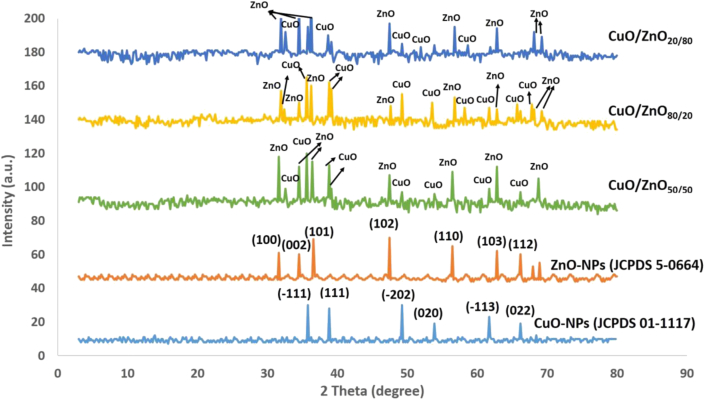
XRD analysis of bio-fabricated CuO-NPs, ZnO-NPs and their nanocomposites at different ratios.

The crystallinity of the bio-fabricated NPs was analyzed by XRD. As shown in Figure 2, the fabricated CuO-NPs showed six defined peaks situated at *2θ* angles of 35.7^o^, 38.9 ^o^, 49.2 ^o^, 53.9 ^o^, 61.8 ^o^ and 66.2 ^o^, which corresponded to (-111), (111), (-202), (020), (-113), and (022) planes, respectively. All the identified peaks of CuO-NPs were assigned according to the JCPDS card number 01-1117 [48]. On the other hand, the major diffraction peaks of ZnO-NPs were observed at *2θ* angles of 31.6 ^o^ (100), 34.5 ^o^ (002), 36.5 ^o^ (101), 47.45 ^o^ (102), 56.55 ^o^ (110), 62.8 ^o^ (103), and 66.14 ^o^ (112) according to the JCPDS card number 5-0664, which indicate the polycrystalline Wurtzite sample structure [30, 49]. Interestingly, the presence of CuO and ZnO peaks in the XRD spectra indicates the successful biosynthesis of the nanocomposites. The size of the crystalline nanoparticle was obtained using Scherrer's equation [44].

The average sizes of the biosynthesized CuO-NPs obtained from the XRD analysis ranged from 3.0 to 10.5 nm, whereas those of the ZnO-NPs ranged from 3.0 to 43.5 nm (Table 1). According to the XRD analysis, the average size of the biosynthesized nanocomposites increased as the ZnO concentration increased. These results are not consistent with those reported by Widiarti et al. [50], where the particle size of CuO/ZnO is decreased after the synthesis of the nanocomposite. The data obtained from XRD show that the average particle size of the fabricated CuO/ZnO_50:50_, CuO/ZnO_20:80,_ and CuO/ZnO_80:20_ were in the range of 3.0–36.1 nm, 3.0–91.0 nm and 3.0–17.0 nm, respectively. This shows that the particle size of the formed composites increased as the contribution portion of ZnO in the nanocomposite formula increased, which confirms the formation of nanocomposites with specific sizes different from those of the individual nanostructures.

• Fourier Transform Infra-Red (FT-IR) Spectroscopy.

**Table 1:** Particle size of CuO, ZnO and their composites with different ratios using XRD analysis.

NPs	Particle size (nm)
CuO	3.0 to 10.5
CuO/ZnO_80:20_	3.0 to 17.3
CuO/ZnO_50:50_	3.0 to 36.1
CuO/ZnO_20:80_	3.0 to 91.4
ZnO	3.0 to 43.5

**Figure 3:**
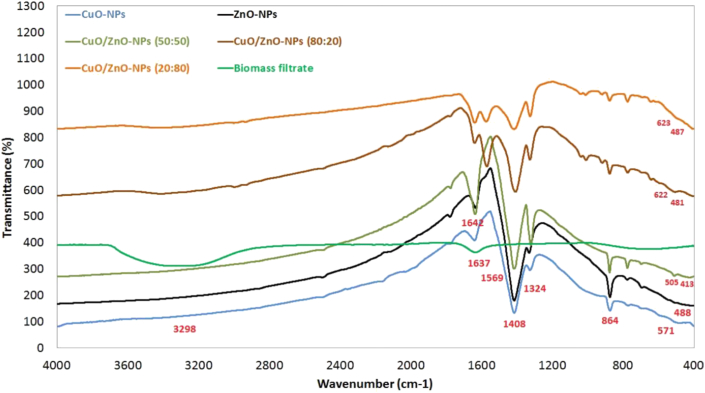
FT-IR spectra of CuO-NPs, ZnO-NPs and their composites at different ratios synthesized by *P. corylophilum* As-1.

FT-IR analysis was conducted to determine the functional groups responsible for the reduction and capping, as well as NPs formation. Figure 3 shows the FT-IR spectra of the biomass filtrate of the *P. corylophilum* As-1, CuO-NPs, ZnO-NPs, and the fabricated nanocomposites in the range of 400–4000 cm^−1^. As shown in the figure, the FT-IR spectra for the fungal biomass filtrate show two distinct peaks at wavelengths of 1637 and 3298 cm^−1^. The peak at 1637 cm^−1^ is related to the C=O stretching vibration bond for biomolecules involved in the fungal biomass filtrate [51, 52], whereas that at 3298 cm^−1^ is related to N–H stretching vibration of aliphatic primary amine [30]. The FT-IR spectra for the NPs and their composites show varying distinct peaks. The appearance of peaks at low wavelengths from 400 to 700 cm^−1^ could be attributed to the successful synthesis of Cu–O and Zn–O in all the samples [53, 54, 55]. In addition, other intense absorption peaks at 3218, 1642, 1569, 1408, 1324 and 864 cm^−1^ are observed in the spectra. The peak at 3218 cm^−1^ corresponds to the O–H stretching group of alcohol and phenols. Also, it may be attributed to N–H asymmetric stretching mode of amines [56]. On the other hand, the observed peak at 1569 cm^−1^ is related to the bending vibrations of amide I band of protein overlapped with N–H stretching. Bands at 1408 and 1324 cm^−1^ are assigned to C–N stretching vibrations of aromatic and aliphatic amines [21], whereas that at 864 cm^−1^ may be attributed to C–H and C=C of alkene [57].

With regard to the functional groups observed in FT-IR spectra, it is obvious that the metabolites present in the biomass filtrate of the *P. corylophilum* As-1 played a major role in the fabrications of the CuO, ZnO and CuO/ZnO composites with stabilized nano-size forms.

• Transmission Electron Microscopy (TEM).

**Figure 4:**
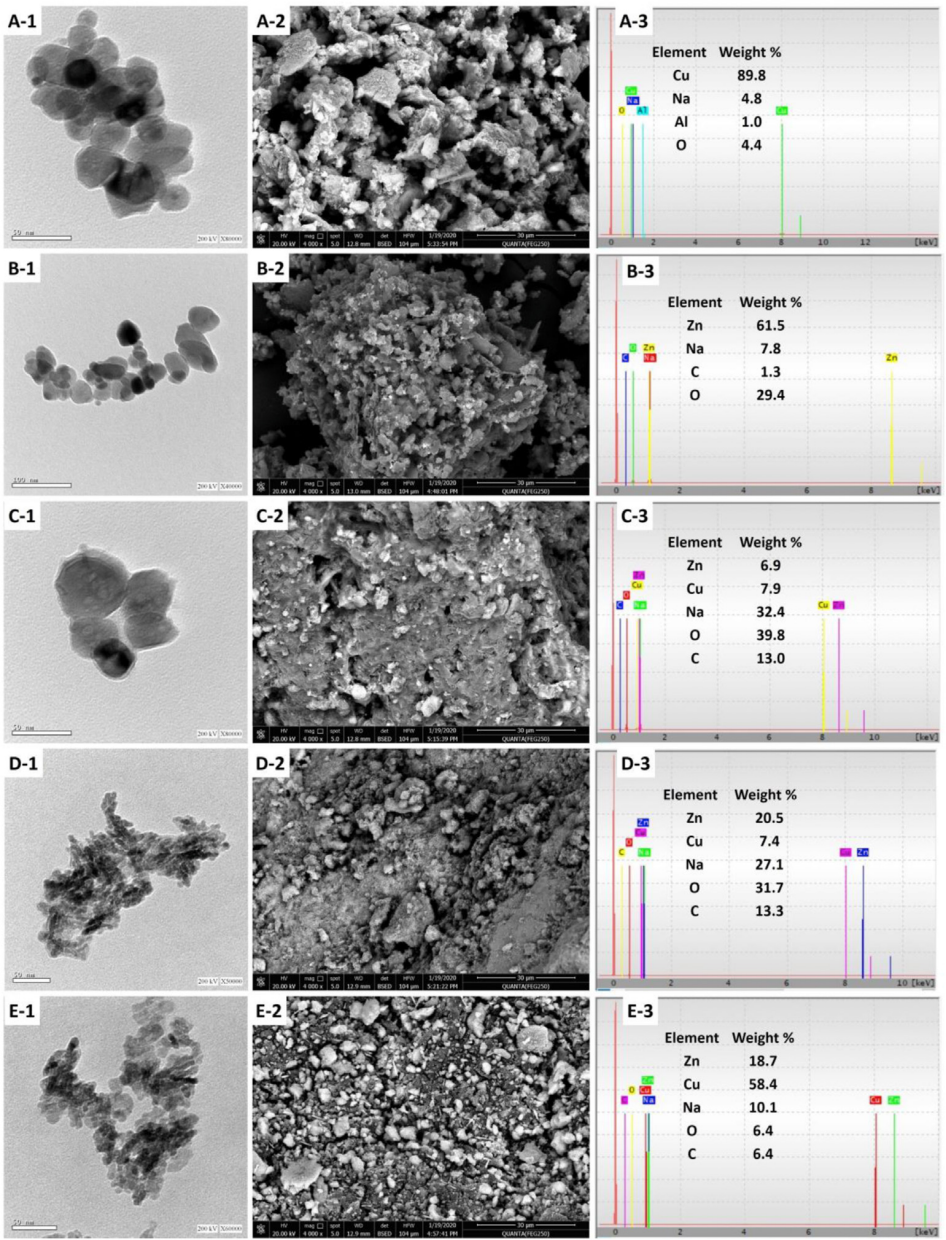
TEM and SEM-EDX analysis of NPs. A-1 to A-3 denotes TEM and SEM-EDX for CuO-NPs; B-1 to B-3 denotes TEM and SEM-EDX for ZnO-NPs; C-1 to C-3 denotes TEM and SEM-EDX for CuO/ZnO_50/50_; D-1 to D-3 denotes TEM and SEM-EDX for CuO/ZnO_20/80_; E-1 to E-3 denotes TEM and SEM-EDX for CuO/ZnO_80/20._

Morphological properties, including size and shape, have critical roles in the biological activities of NPs. Therefore, the biosynthesized CuO-NPs, ZnO-NPs, and CuO/ZnO nanocomposites with different ratios (50/50; 20/80 and 80/20) were investigated *via* TEM analysis to determine their respective sizes and shapes (Figure 4 A-1 to E-1). The TEM images confirm the successful biosynthesis of nano-spherical shapes with different sizes, ranging from 23.8 to 45.0 nm and 9.20–51.73 nm for individual nanoparticles of the CuO-NPs and ZnO-NPs, respectively. Also, the CuO/ZnO_50/50_ nanocomposite showed spherical particles with an average size of 30.18–55.63 nm. The nanocomposites of the other ratios exhibited different shapes ranging between spherical, ellipsoidal and cylinder, as in CuO/ZnO_20/80_ and CuO/ZnO_80/20_ nanocomposites, and also showed varying sizes ranging from 5.0 to 13.2 nm and 6.8–19.2 nm, respectively. The condensed nanocomposites that appeared in the TEM image could be related to the high composition of the two synthesized NPs [58]. The TEM images also revealed that CuO/ZnO_20/80,_ and CuO/ZnO_80/20_ had smaller particle sizes than the pure NPs and CuO/ZnO_50/50_. The obtained data are in agreement with that of Sisk and Hope-Weeks [59] and Gao et al [60], who reported that the synthesized composites differ in morphology and their size from the individual structure under the same production conditions.

• Scanning Electron Microscopy (SEM-EDX)

The surface morphology of the biosynthesized NPs was investigated using an SEM connected with X-ray for semi-quantitative elemental compositions. As shown in Figure 4 (A-2 to E-2), nearly spherical NPs with little agglomerations were obtained. These agglomerations could be attributed to the high tendency of NPs to form large clusters [61]. On the other hand, EDX analysis confirmed the successful synthesis of CuO, ZnO, and their composites with different CuO/ZnO ratios (Figure 4 A-3 to E-3). According to the EDX spectra, the CuO-NPs contain Cu, O, Na, and Al with weight percentages of 89.8%, 4.4%, 4.8%, and 1.0 %, respectively, and the ZnO-NPs contain Zn, O, Na, and C with weight percentages of 61.5%, 29.4%, 7.8%, and 1.3 %, respectively. It was also shown that Cu and Zn ions were in high concentrations followed by oxygen. Also, EDX spectra of CuO/ZnO-NPs_50/50_ confirmed the formation of hybrid nanocomposite at similar weight percentages of 7.9% and 6.9% for CuO and ZnO, respectively. In the same regard, the weight percentage of Cu, Zn, O, C, and Na in the composite CuO/ZnO_20/80_ was found to be 7.4%, 20.5%, 31.7%, 13.3%, and 27.1%, respectively, whereas it was 58.4%, 18.7%, 6.4%, 6.4%, and 10.1%_,_ respectively, for the CuO/ZnO_80/20_ composite. The presence of distinct peaks of C and Na may be attributed to the hydrolysis of the capping fungal metabolites containing proteins, sugars, amino groups, and carbohydrates by X-ray emissions [42, 62, 63]. The obtained data are in good agreement with that of Mohammadi-Aloucheh et al [42], where it was reported that the weight percentage of Zn, Cu, O, and C in a composite of ZnO/CuO (5%) synthesized using a leaf extract of *Mentha longifolia* was 72.1%, 4.1%, 21.4%, and 2.4%, respectively, and for the composite ZnO/CuO (10%), it was 73.2%, 4.3%, 19.2%, and 2.3% respectively.

• X-ray Photoelectron Spectroscopy (XPS) Analysis

**Figure 5:**
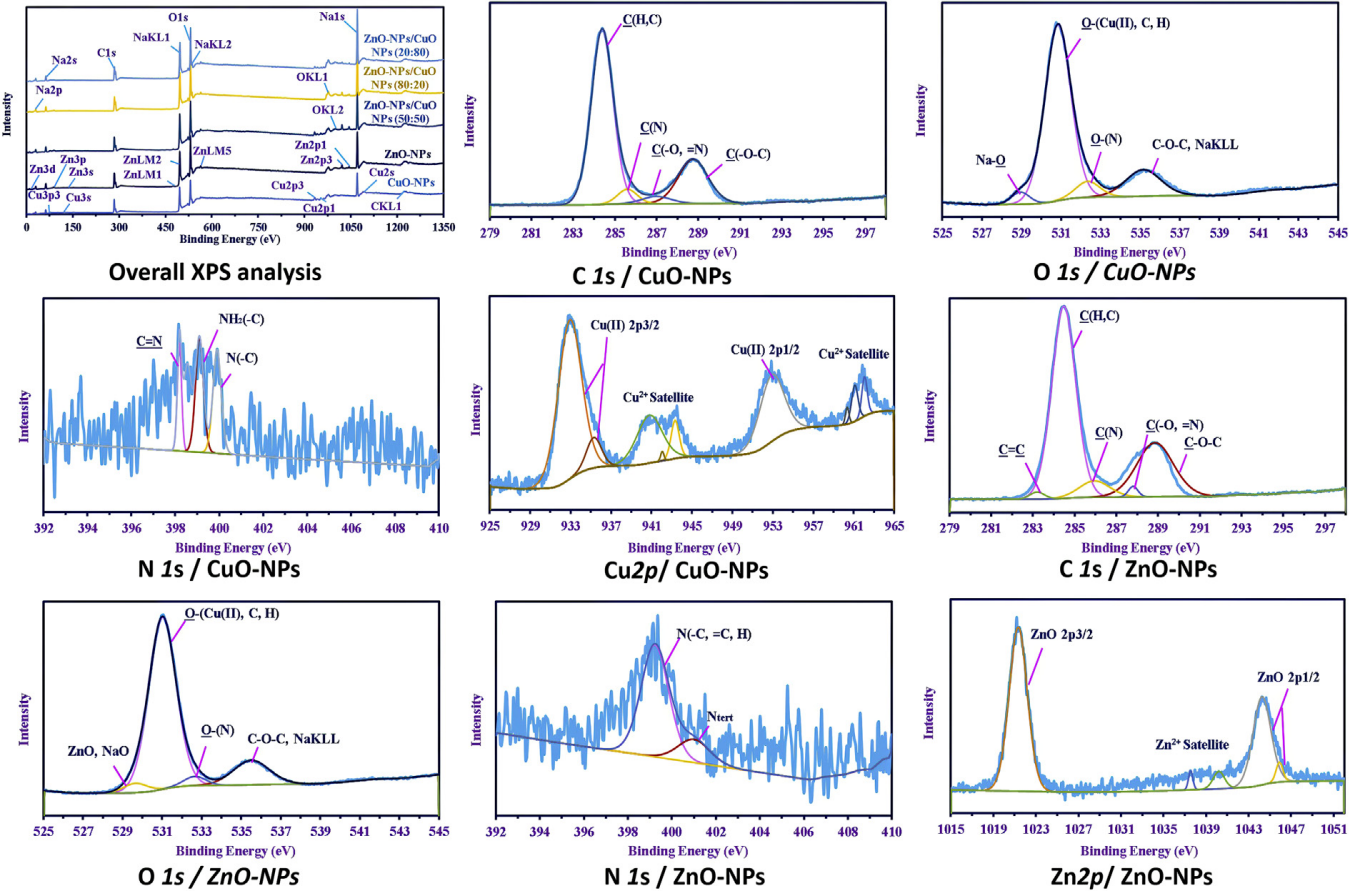
Overview and Core XPS analysis for CuO-NPs and ZnO-NPs synthesized by *P. corylophilum* As-1.

The XPS analysis confirmed the presence of Cu and Zn with varying binding energies according to the type of product. The binding energies of Cu and Zn as individual metals in the CuO-NPs and ZnO-NPs were investigated. Cu was found on several binding energy levels, including *3p3, 2p1, 2p3, 2s,* and *3s*, whereas Zn in ZnO-NPs was found on *3d, 3p, 3s, LM1, LM2, LM5, 2p3,* and *2p1*. The intensity of these peaks for different ratios shows the real quantities of these metals, as in CuO/ZnO_50/50._ The presence of both Cu and Zn was confirmed in the nanocomposite, however, as the ratios changed, some peaks disappeared. Besides this, other peaks related to hydrocarbons and medial components were observed as Na, which found as Na (*2p, 2s, 1s, KL1,* and *KL2*), O (*2s, 1s, KL1* and *KL2*), and C1s (*1s,* and *KL1*) (Figure 5).

**Figure 6:**
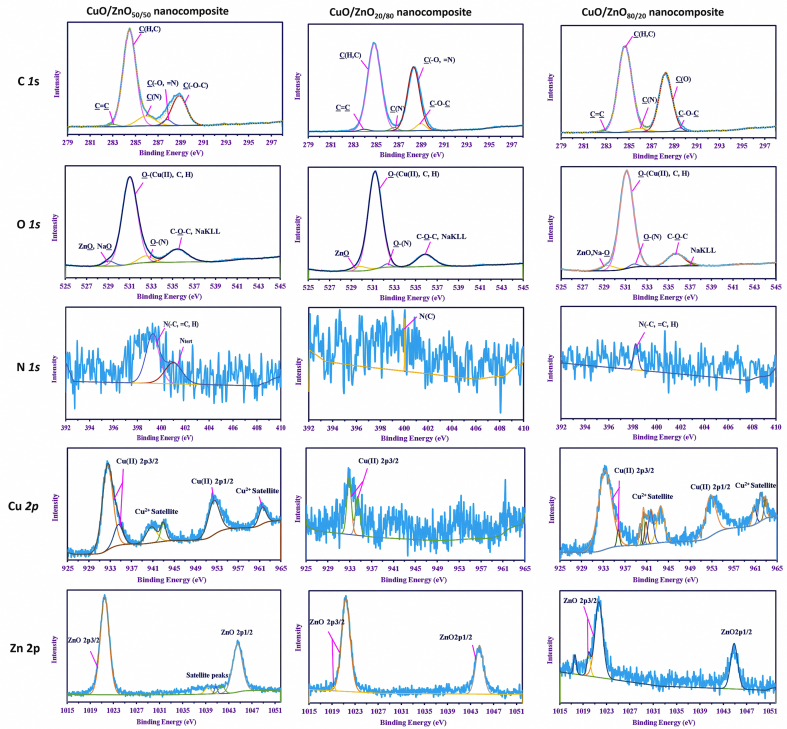
Core XPS analysis for CuO/ZnO nanocomposites with different ratios synthesized by *P. corylophilum* As-1.

The data represented in Figures 5 and 6 show the HRES of the interesting peaks that reflect a variation in each product. For example, different splitting peaks were observed for C *1s* depending on the product, where four peaks corresponding to C(H,C), C(N), C(O,=N), and C–O–C were shown in the CuO-NPs at 284.39 eV, 285.6 eV, 287 eV, and 288.75 eV, respectively [64, 65, 66]. These peaks were also observed in ZnO-NPs but with upshifts in energies (284.48 eV, 285.95 eV, 287.35 eV, and 288.85 eV, respectively), besides other new peaks assigned to C=C at 283.2 eV [67]. This phenomenon is due to the effect of metal ions on the hydrocarbons. The nanocomposites showed C=C on all the products. The peaks of C=C, C(H,C), C(N), C(O=N), and C–O–C appeared at 283 eV, 284.48 eV, 285.95 eV, 287.8 eV, 288.9 eV, respectively, in the CuO/ZnO_50/50_ nanocomposite. These peaks were shifted to higher/lower binding energies as the ratio of Zn increased/decreased, and they appeared at 283.95 eV, 284.83 eV, 286.6 eV, 288.23 eV, and 288.95 eV in the CuO/ZnO_20/80_ nanocomposite. For CuO/ZnO_80/20_, the peaks were shifted to 282.75 eV, 284.65 eV, 285.95 eV, 288.25 eV, and 289.5 eV, respectively.

O *1s* is deconvoluted into four peaks of the all series of the precipitates, which confirms the presence of metal as oxides (i. e. CuO and ZnO), sharing with Na–O (in Zn precipitate) and Na *KLL,* which overlapped with C–O–C [47]. The obtained results indicate that the product was contaminated with Na in addition to the constituents of the hydrocarbons as by the peaks of O–N, C–O–C, O–H, and O–C [68, 69]. The obtained results are parallel to that obtained from the FT-IR analysis, which showed a broad band at around 3300 cm^−1^ attributed to OH overlapped with NH. On the other hand, the presence of the C–O band at around 860 cm^−1^ was observed in the all the products. Also, the FT-IR showed OH out of the plane at around 740 cm^−1^ [70, 71, 72].

N *1s* showed varying intensities among different products. A high ratio of Cu resulted in higher intensity of nitrogen. However, the products with pure Cu, as in CuO-NPs, showed three peaks assigned to C≡N, NH_2_(C), and N_tert,_ which appeared at 398.19 eV, 399.1 eV, and 399.9 eV, respectively. The first two peaks in the ZnO-NPs product, which appeared at 399.23 eV, overlapped with little shift and increasing AF%. However, the energy shifted higher in N_tert,_ where the peaks were shown at 401.0 eV [73]. Both peaks were observed in the composite with a ratio of 50/50, where they appeared at 399.26 eV and 401.0 eV, respectively. Only one peak exhibited low intensity at different binding energies for the other ratios that have appeared at 400.04 eV for N_tert_ in CuO/ZnO_20:80_ ratio, while the peak at 398.25 eV in CuO/ZnO_80/20_ ratio for overlapping C≡N and NH_2_(C) [74, 75].

The intensity of Cu (II) and Zn (II) was dependent on the ratio represented in the products, whereas the Cu *2p* and Zn *2p* were the most familiar binding energies of the two elements.

Cu (II) was split according to the type of the product in the CuO-NPs (represent as the lonely elements) that was deconvoluted into eight peaks at 932.92 eV and 935.3 eV for Cu(II) *2p* 3/2. The peak at 952.85 eV was assigned to Cu(II) *2p* 1/2, whereas the satellite peaks were observed at 940.8 eV, 942.0 eV, 943.5 eV, 960.35 eV, and 961.85 eV. As discussed above in the data for O *1s* and Cu *2p,* Cu was found as divalent ion and mainly in oxide form. Zn *2p* of ZnO-NPs was split into five peaks at 1021.34 eV (the main peak with AF% of 56.03%) for ZnO *2p* 3/2. The next main peaks appeared at 1044.25 eV (AF% = 30.06%), and 1045.9 eV (AF% = 2.61%) for ZnO *2p* 1/2. The other peaks at 1037.55 eV and 1040.2 eV with AF% of 4.57% and 6.73%, respectively, were assigned to satellites peaks, indicating its presence as Zn(II) oxide.

These peaks were found with little shifts, and some of them disappeared as the concentration changed. For the 50/50 ratio, six peaks were observed for Cu *1s* at 932.51 eV, and the peak at 934.55 eV for Cu(II) *2p* 3/2, whereas that corresponding to Cu(II) *2p* 1/2 appeared at 952.2 eV and the satellite peaks at 940.8 eV, 943.4 eV, and 961.45 eV. On the other hand, Zn *2p* was split into five peaks shown at 1021.51 eV for ZnO *2p* 3/2, 1044.5 eV for ZnO *2p* 1/2 with AF% of 56.41% and 33.92%, respectively. Peaks at 1039.3 eV, 1040.7 eV, and 1041.75 eV, which are attributed to Zn^2+^ satellites, where also observed. This is similar to those of the individual nanoparticles but with little difference in the shifts. In addition, low-intensities peaks disappeared in this case, and the main peaks appeared with a little shift.

For ZnO/CuO_80:20,_ the external shape and splitting peaks were different from those of other composites. Two low-intensity peaks were observed for Cu *2p* 3/2 at 932.87 eV and 934.45 eV, and Zn *2p* was deconvoluted into three peaks. ZnO *2p* 3/2 exhibited two deconstructed peaks appeared at 1021.39 eV and 1019.3 eV, and ZnO *2p* 1/2 appeared at 1044.55 eV, which indicates a higher percentage of Zn than Cu. Contrary to the products of ZnO/CuO_20:80,_ the results show that Cu *2p* was deconstructed into 11 individual peaks at 933.3 eV and 935.75 eV for Cu(II) *2p* 3/2, peak 952.75 eV for Cu(II) *2p* 1/2, and others for Cu(II) satellite, which appeared at 939.75 eV, 940.35 eV, 940.85 eV, 941.75 eV, 943.55 eV, 960.8 eV, 962.0 eV, and 962.85 eV. It was also obtained that Zn *2p* was split into three peaks shown at 1021.69 eV and 1019.95 eV for ZnO *2p* 3/2, and 1044.85 eV for ZnO *2p* 1/2. From these results, we confirmed that, the metal ions were present in different ratios and existed as M (II) oxides (M = Zn or Cu).

### 3.3 Photo-degradation efficiency

• Optical properties (Diffused reflectance spectrum).

**Figure 7:**
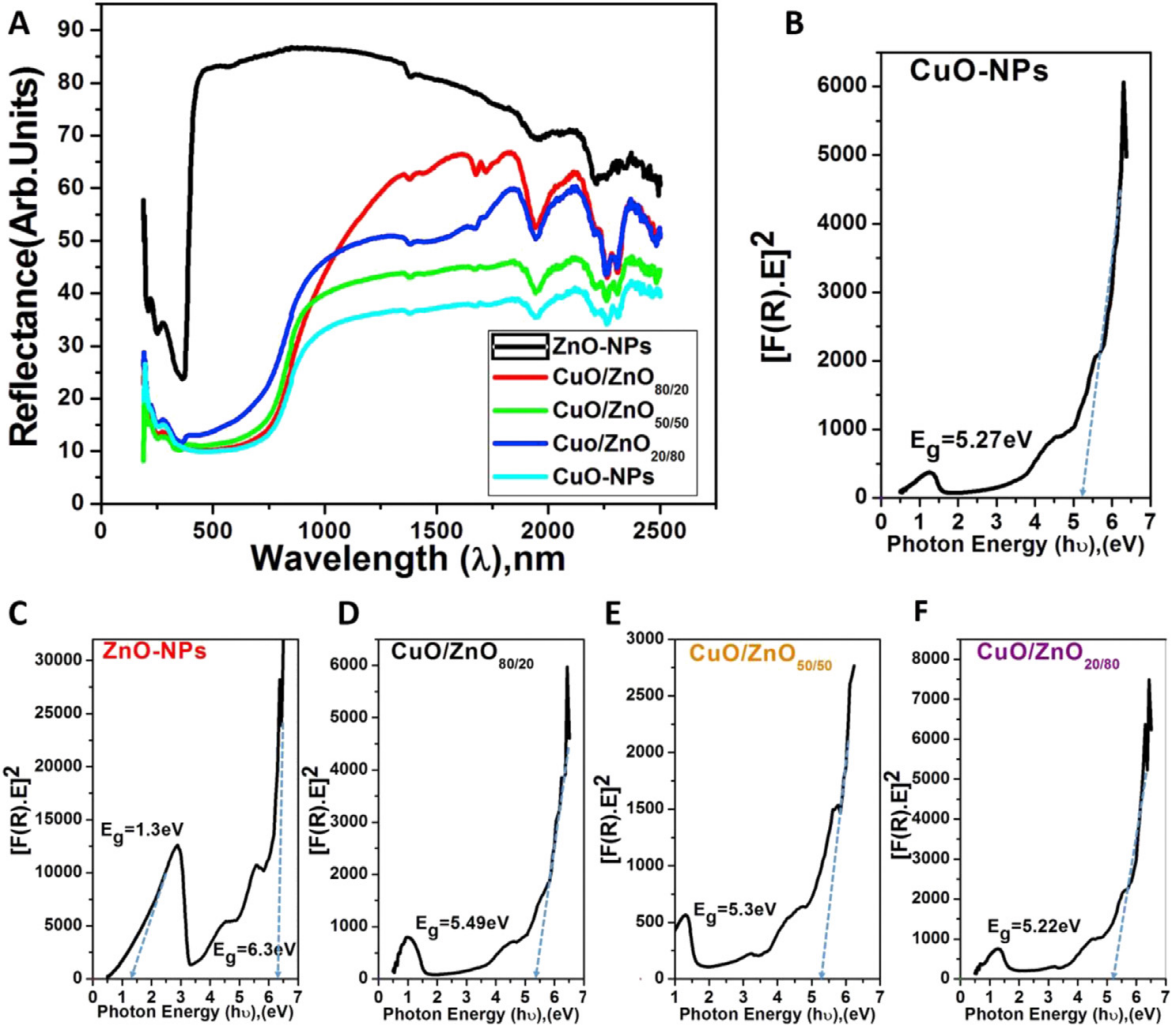
(A) denote diffuse reflection spectra for CuO-NPs, ZnO-NPs and their composites; (B, C, D, E and F) denotes plot of [F(R)∗E] ^2^ against Photon energy (hυ) for all synthesized NPs.

The optical diffused reflection spectra for the dried biosynthesized NPs powder was analyzed using a UV-vis spectrophotometer. Figure 7A shows the optical reflection spectra of CuO-NPs, ZnO-NPs, CuO/ZnO_80/20_, CuO/ZnO_50/50,_ and CuO/ZnO_20/80_. The bandgap and induced activation of light toward the visible light region or the transfer photo-activated electrons using a photo-sensitizer for the absorbed light in the visible region increase the electrons-hole separation of accelerate the transfer of interfacial energy, thus causing efficient degradation of the dye and photo-electrochemical activity toward water splitting.

The optical bandgap energy of the samples under investigation was calculated from the optical reflectance data using the Kubelka-Munk model as reported by Zhu et al [76] and Parthibavarman et al [77], which is shown below.

(4) $$\text{FR}=\frac{{\left(1-\text{R}\right)}^{2}}{2\text{R}}$$

where R is the diffused reflectance. The estimated values of the bandgap energies are depicted in Figure 7 (B to F), which shows the intercept of the straight line on the photon energy axis from the plot of [F(R)∗E]^2^ against photon energy (hυ). It shows optical bandgaps of 6.32 eV, 5.49 eV, 5.3 eV, 5.22 eV, and 5.27 eV for ZnO, CuO/ZnO_80/20_, CuO/ZnO_50/50_, CuO/ZnO_20/80,_ and CuO, respectively. These bandgaps are relatively high, especially for ZnO-NPs, which confirms that an increase in CuO ratio in the nanocomposites results in a tangible decrease in the bandgap.

• Adsorption and photocatalytic behavior

**Figure 8:**
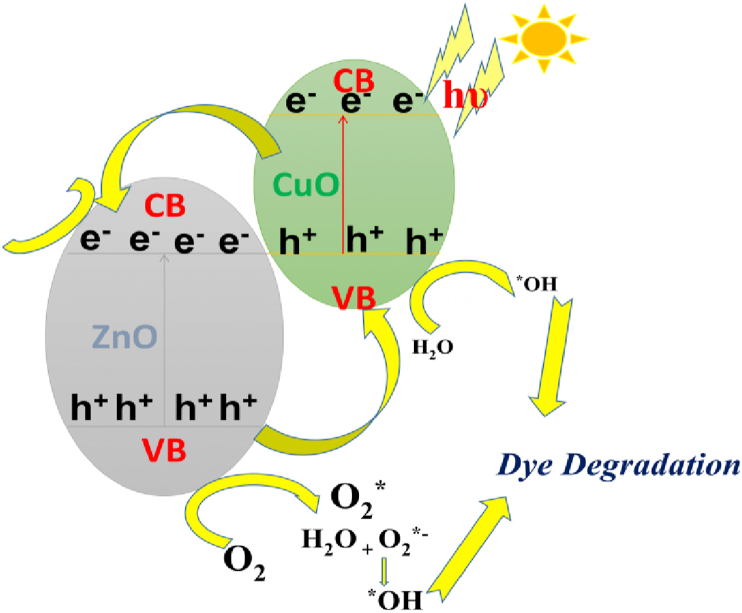
The photo-degradation mechanism for Methylene Blue dye in the presence of Cuo@Zno nanocomposite

The photocatalytic mechanism of semiconductors, in the case of the light-absorbing species, is semiconducting material. The electronic structures of semiconductor materials show two-level bands; the highest occupied band filled with electrons is referred to as the valence band (VB), and the lowest unoccupied band, referred to as the conduction band (CB). The forbidden region between the two bands measures the bandgap energy (E_g_). Ultra-bandgap illumination of such semiconductor materials produces electron (e^−^)-hole (h^+^) pair, which results in the photocatalysis of the semiconductor particles up to the ultra-bandgap excitation in an aqueous solution containing dissolved oxygen and oxidizable pollutant (Figure 8) [78, 79, 80]. The process involves:

(1) Electron-hole recombination in bulk.
(2) Electron-hole recombination at the surface.
(3) Direct or indirect reduction of oxygen.

The transition of the electrons possess high activity to interact with oxygen groups on the surface of the CuO/ZnO nanocomposites and with H_2_O molecules to generate radical anions, including reactive oxygen species (ROS), superoxide anion radical scavengers (O_2_**^∗^**^**-**^), and hydroxyl (^∗^OH) as follow:

(5) $$\text{C}uO/ZnO+hv\to CuO\text{/}ZnO\left[h_{\left(VB\right)}^{+}+e_{\left(CB\right)}^{-}\right]$$

(6) $$CuO/ZnO\left[h_{\left(VB\right)}^{+}+e_{\left(CB\right)}^{-}\right]\to CuO\left(h^{+}\right)+ZnO\left(e^{-}\right)$$

(7) $$ZnO\left(e^{-}\right)+O_{2}\to {\text{O}}_{2}^{\text{∗}-}$$

(8) *CuO*(*h*^*+*^)*+H*_*2*_*O→OH∗+H*^*+*^

(9) *OH∗+Dye M.B.→Degraded products*

**Figure 9:**
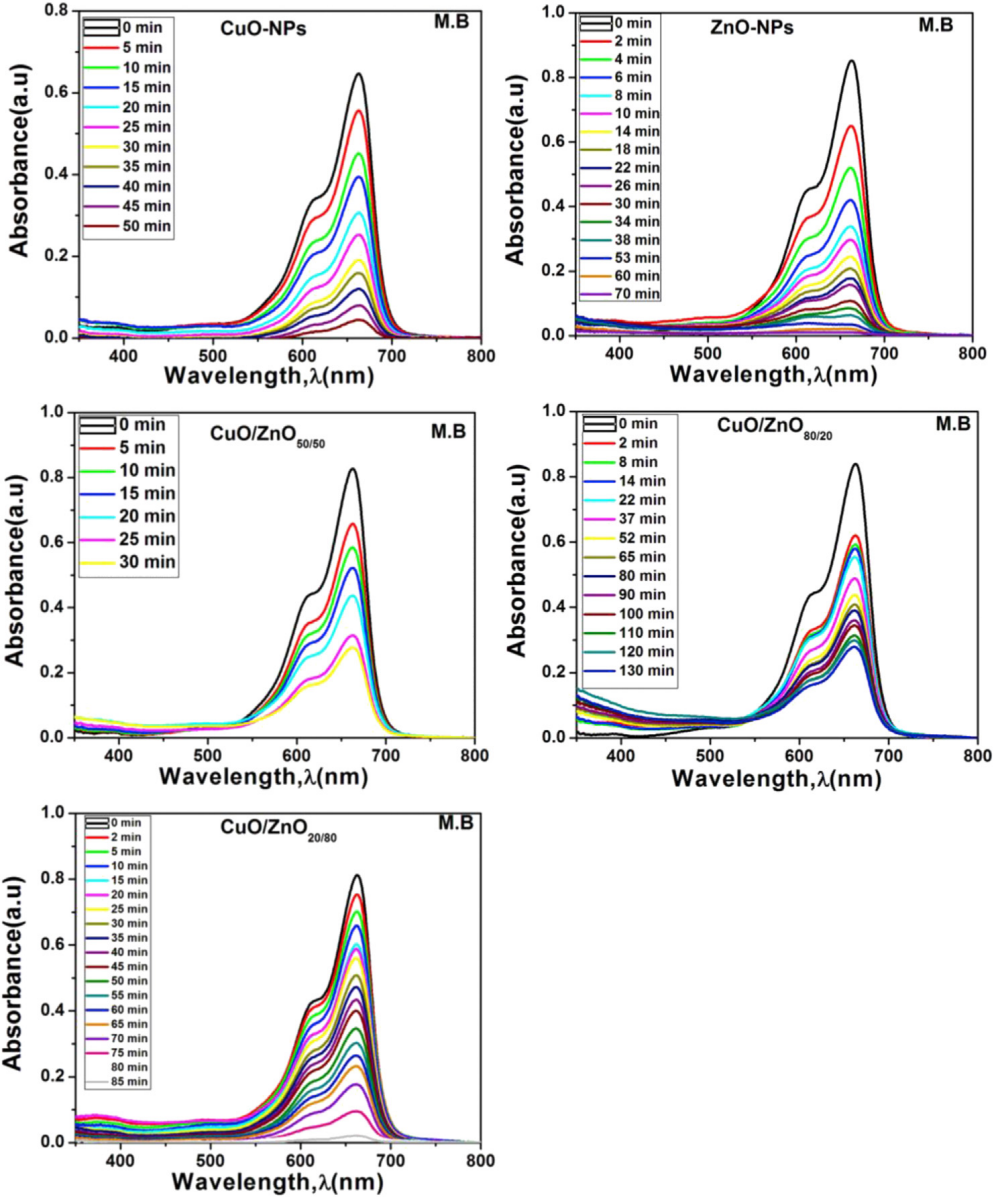
UV-vis spectra of methylene blue dye for ZnO-NPs, CuO-NPs, CuO/ZnO_50/50_, CuO/ZnO_80/20_, and CuO/ZnO_20/80_.

The UV-vis absorption spectra of the organic dye (MB) for ZnO, CuO/ZnO_80/20_, CuO/ZnO_50/50_, CuO/ZnO_20/80,_ and CuO in the dark state at different times of reduction by the light lamp, especially at the cationic MB, show a strong absorption band at 663 nm and another shoulder band at 614 nm. On the other hand, it was observed that for all samples, the intensity of the adsorption peaks decreased gradually as the time of degradation increased, and the MB underwent complete degradation at 70.0, 50.0, 30.0, 130.0, and 85.0 min after light absorption (Figure 9). Consequently, the azo bonds and aromatic rings of the MB molecules were destroyed under visible light absorption in the presence of the photocatalyst [81, 82]. As a result, the rate of degradation of MB increased as the ZnO ratio in nanocomposites increased. The removal efficiency of about 97% was achieved by CuO/ZnO_20/80_ after 85.0 min of irradiation, whereas CuO/ZnO_80/20_ nanocomposite achieved a maximum removal efficiency of 65% after 130.0 min. The removal efficiency of CuO/ZnO_20/80_ nanocomposite was much closer to that of ZnO-NPs. The nanocomposites exhibited improved characteristics, including high thermal stability and low photo-corrosion compared with the individual nanoparticles.

**Figure 10:**
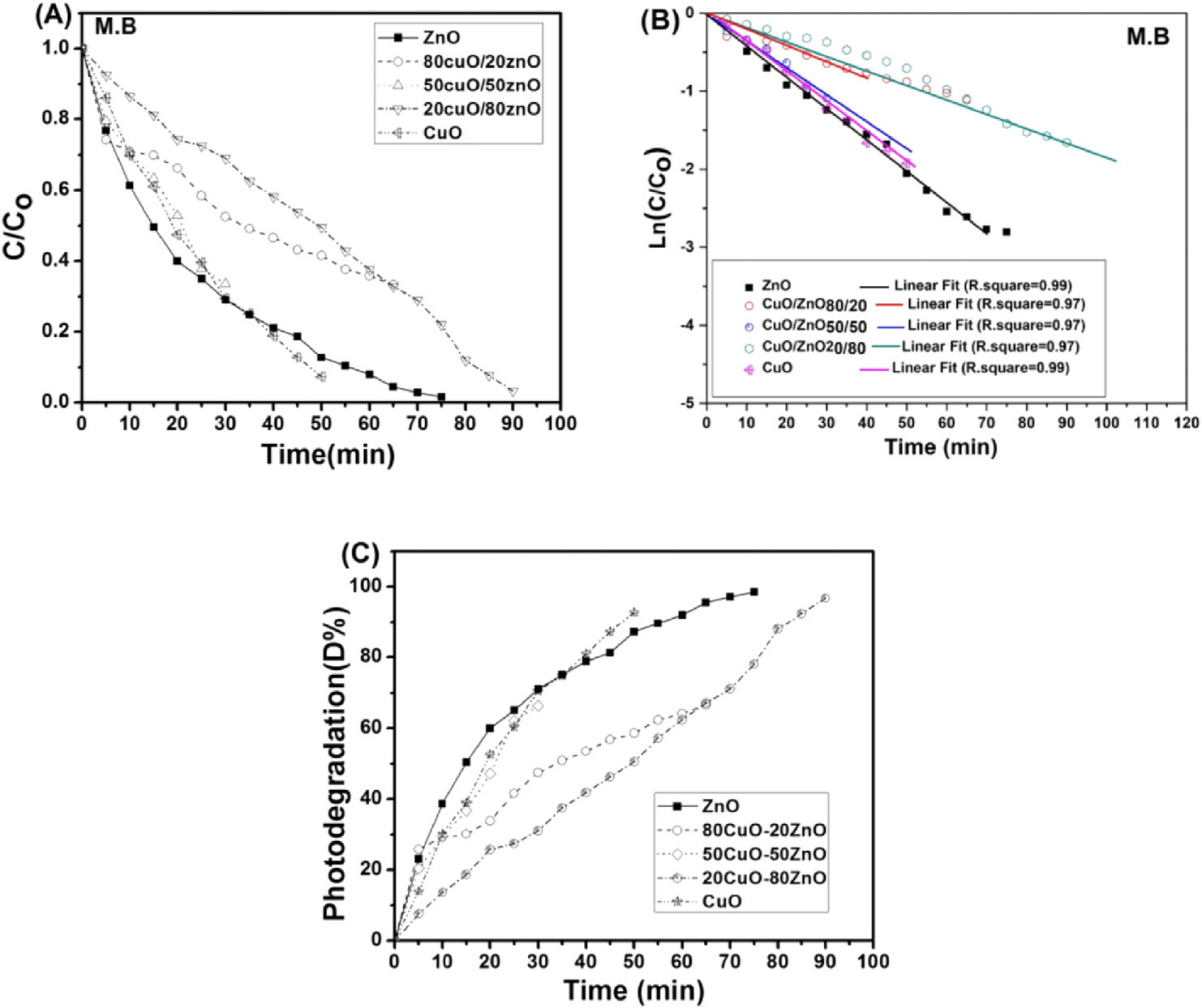
(A) Time degradation curves, (B) plot of ln(C/C0) against time (min), (C) Photo degradation (D%) *Vs* time (min) of visible light.

The Longmuir-Hinshelwood model was employed to clarify the photo-degradation kinetic of the catalyzed reactions (Figure 10). In a simple form, the model for the apparent pseudo-first-order is expressed as follow:

(10) $$\text{ln}\left(\frac{C}{C_{\text{0}}}\right)=-K_{app}t$$

where (*K*_app_) is the apparent pseudo-first-order constant (min^−1^). The plot of ln(C/C0) against time is a linear function with the slope equal to (*K*_app_)**.**

**Figure 11:**
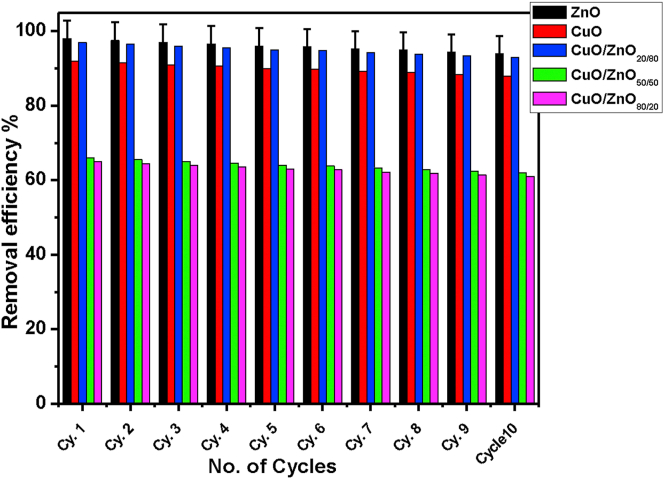
The reusability of nanocomposites after 10 cycles of running.

The stability and reusability of any photocatalyst are important in continuous adsorption processes. The CuO-NPs, ZnO-NPs, CuO/ZnO_80/20_, CuO/ZnO_50/50,_ and CuO/ZnO_20/80_ nanocomposites were subjected to cyclic decomposition tests, and at the end of each cycle, the hybrid was separated from the MB solution and added to freshly prepare 10 mg L^−1^ of the solution for subsequent degradation test. Figure 11, shows that there was no apparent reduction in the photo-degradation efficiency even after repeated use. This illustration is based on the structural stability of the hybrid photocatalyst coupled with the catalytic activity of semiconductor nanoparticles [83]. It was observed that the reduction in the rate of photocatalytic degradation after the 10^th^ cycles was less than 4% (from 98% to 94%) for ZnO. On the other hand, for CuO/ZnO_80/20_, CuO/ZnO_50/50_, CuO/ZnO_20/80_ and CuO it was reduced to from 65%–61%, 66%–62%, 97%–93%, and 92%–88%, respectively.

## 4 Conclusion

In this study, green nano-biotechnology was employed to prepare photocatalyst nanocomposites (nano-photocatalyst). A combination of two semiconductors could induce mutual properties that would improve the activity of the hybrid composites compared to the individual materials. Herein, one of the most common photocatalyst, CuO/ZnO nanocomposite, which has higher thermally stability than either of the ZnO and CuO nanoparticles, was biosynthesized by cultivating copper and zinc precursors with biomass filtrate of *Penicillium corylophilum* strain As-1. The obtained CuO/ZnO nanocomposites were optimized and characterized by FT-IR, XRD, SEM-EDX, XPS, and TEM to obtain their chemical and morphological structures. The obtained results reveal that CuO/ZnO nanocomposites with different ratios (CuO/ZnO_50/50_, CuO/ZnO_80/20,_ and CuO/ZnO_20/80_) were successfully synthesized. It was obtained that an increase in the ZnO ratio in the obtained composites resulted in an increase in the average particle size of the composites. Furthermore, the photocatalytic degradation efficiency also increased with an increase in the ZnO ratio, as observed in CuO/ZnO_20/80_. Notably, the composite achieved a removal efficiency of 97 % for MB dye after 85.0 min of irradiation in by visible light.

## Declarations

### Author contribution statement

Amr Fouda, Salem S. Salem, Ahmed R. Wassel, Mohammed F. Hamza, Th. I. Shaheen: Conceived and designed the experiments; Performed the experiments; Analyzed and interpreted the data; Contributed reagents, materials, analysis tools or data; Wrote the paper.

### Funding statement

This research did not receive any specific grant from funding agencies in the public, commercial, or not-for-profit sectors.

### Competing interest statement

The authors declare no conflict of interest.

### Additional information

The data used to support the findings of this study are available from the corresponding author upon request.
